# All-Solid-State Textile Batteries Made from Nano-Emulsion Conducting Polymer Inks for Wearable Electronics

**DOI:** 10.3390/nano2030268

**Published:** 2012-08-13

**Authors:** Di Wei, Darryl Cotton, Tapani Ryhänen

**Affiliations:** Nokia Research Centre, Broers Building, 21 JJ Thomson Av., CB3 0FA, Cambridge, UK; Email: daryl.cotton@nokia.com (D.C.); tapani.ryhanen@nokia.com (T.R.)

**Keywords:** polymer battery, all-solid-state textile battery, PEDOT, conducting polymer, nano-emulsion ink

## Abstract

A rollable and all-solid-state textile lithium battery based on fabric matrix and polymer electrolyte that allows flexibility and fast-charging capability is reported. When immerged into poly(3,4-ethylenedioxythiophene) (PEDOT) nano-emulsion inks, an insulating fabric is converted into a conductive battery electrode for a fully solid state lithium battery with the highest specific energy capacity of 68 mAh/g. This is superior to most of the solid-state conducting polymer primary and/or secondary batteries reported. The bending radius of such a textile battery is less than 1.5 mm while lightening up an LED. This new material combination and inherent flexibility is well suited to provide an energy source for future wearable and woven electronics.

## 1. Introduction

Lithium ion batteries have been used in almost all mobile devices including PDAs, laptops and mobile phones. Development of organic displays and flexible OLEDs show promise in enabling a device which can be bent or twisted. However, these new form factors also require a pliable battery with a high specific energy capacity. Current lithium ion battery electrodes are made by coating lithium metal oxide slurries onto charge collectors. This method is not suitable in applications that require mechanical flexibility, due to the brittle nature of slurry coatings when bent or stretched.

A lot of work has been made on developing stretchable conducting yarns [1] and textile electrodes for supercapacitors [2,3,4]. The basic strategy is to apply a coating of carbon nanotube (CNT)/MnO_2_ solutions onto cotton textiles as electrodes. It is generally not easy to reproduce/control the uniformity of CNT coating and the potential toxicity of CNTs [5] also limits their application in wearable electronics. On the other hand, conducting polymers have been explored as electrodes for both supercapacitors [6,7] and batteries [8], but one of their main limitations lies in the poor adhesion to the charge collecting metal foils when used in batteries. In this paper, a cost-effective and binder-free method is developed by coating high-surface area, flexible and stretchable textiles with conducting polymer nano-emulsion inks. The polymer nano-emulsion inks can be quickly soaked into the textile matrix and directly used as cathodes in rechargeable lithium batteries. 

Conducting polymers, also known as conjugated polymers including polyaniline, polypyrole, polythiophene *etc.*, consist of alternating single and double bonds to create an extended π-network [9]. Conductivity of such polymers falls in the range of semiconductors, and some of them can be made as conducting as metals when doped by photochemical, chemical or electrochemical methods [10]. Lithium batteries can be made from various conducting polymers. All-solid-state Li/polyaniline batteries offered specific energy of 50 Wh/kg [11]. Specific energies in range of 40–60 Wh/kg have been predicted for packed Li/polypyrrole cells with optimized design. When a poly(ethylene oxide) (PEO) polymer electrolyte was used, the specific energy of an all-solid-state Li/polypyrrole battery was reported to be 55 Wh/kg [12]. Among these conducting polymers a derivative of polythiophene, poly(3,4-ethylenedioxythiophene) (PEDOT) had been generally used as the charge collecting layers in organic electronics [13]. Especially when it is used in organic photovoltaics, charge collection efficiency, photocurrent efficiency and device performance were enhanced significantly. Such enhancing effect of PEDOT may be partially due to a better Ohmic contact with the lithium metal.

## 2. Results and Discussion

**Figure 1:**
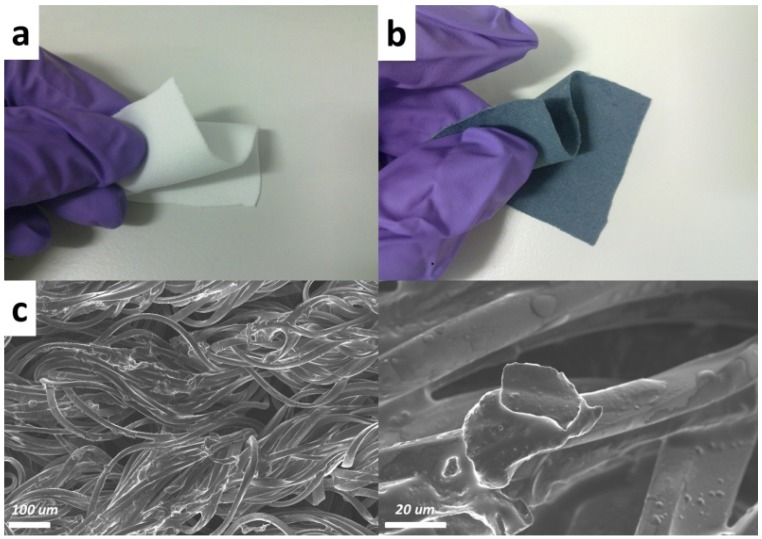
Picture of (**a**) polyester/cellulose fabric;(**b**) poly(3,4-ethylenedioxythiophene) (PEDOT) soaked polyester/cellulose fabric as textile battery electrodes and (**c**) scanning electron microscope (SEM) images of the soaked textile.

In our experiments, strong and durable polyester/cellulose hydroentangled nonwoven fabric cloth embedded with PEDOT nano-emulsion inks was used as a battery cathode material. The textile is exceptionally absorbent in terms of weight-to-capacity ratio and works very well as a scaffold for conducting polymer (PEDOT) nano-emulsion inks. Figure 1a shows the intrinsic polyester/cellulose fabric and Figure 1b shows PEDOT soaked textile material. The conductive textile was prepared by pipetting PEDOT nano-emulsion inks onto the textile and baking at 100 °C for 2 h. The sheet resistance of the textile was measured with a RCHEK 4-point meter before and after soaking and confirmed it changes from insulator to a conductor with a sheet resistance of approximately 160 Ohm/sq. This is comparable with the electrochemical deposited polypyrrole (171 Ohm/sq) [14]. The embedded PEDOT functions both as charge collector and electro-active material in our battery design. 

The original fabric (Figure 1a) is intrinsically insulating, thus only the morphology of the conducting fabric soaked with PEDOT (Figure 1b) was characterized with a scanning electron microscope (SEM, FEI/Philips XL30) in high vacuum mode (3.5 × 10^−6^mbar). Figure 1c shows that the PEDOT is coated evenly on the fibres of the polyester/cellulose textile. Diameters of such tangled spaghetti textile fibers range from 10 to 20 µm. 

**Figure 2:**
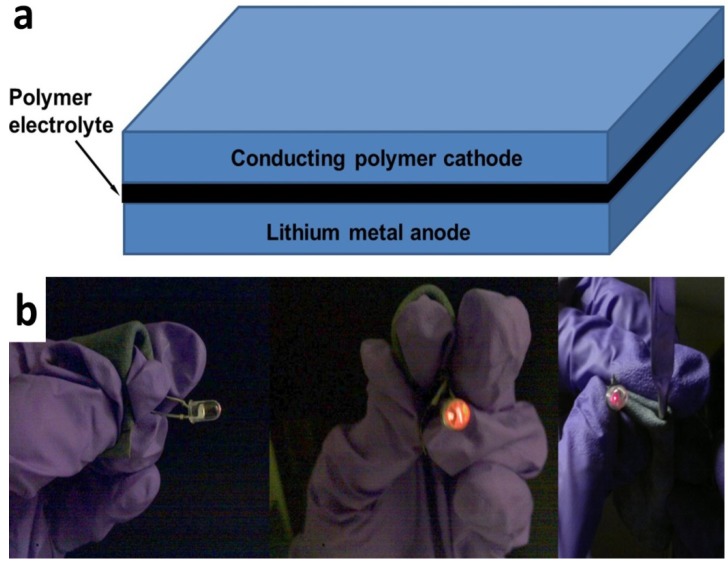
(**a**) Structure of the textile battery and (**b**) flexible and fully rollable batteries made from PEDOT soaked textile that can light up an LED.

The textile batteries were constructed from a solid polymer electrolyte sandwiched between a lithium foil anode and PEDOT textile cathode as illustrated in Figure 2a. The polymer electrolyte was composed of high molecular weight poly(ethylene glycol) borate ester [15]. Performance of such polymer electrolytes is comparable with conventional liquid organic electrolytes at room temperature. To make it more flexible the separator/ polymer electrolyte can either be a fabric separator as reported in reference [4] or a stretchable gel polymer electrolyte based on monovalent acrylate polymer matrices [16]. Open circuit voltage of such a textile battery based on PEDOT is over 2.8 V, in contrast, voltages of the batteries based on other conducting polymers such as polypyrrole are around 1 V [14,17]. Thus a reliable, flexible and rollable battery from PEDOT soaked textile can be used to light up an LED due to its high open circuit voltage, even with bending radius smaller than 1.5 mm. Figure 2b shows the battery wrapped around a spatula with a radius of < 1.5 mm and the whole textile battery can also be totally wrapped up into a roll in operation. It should also be noticed that such battery is still in full operation after 100 times bending test. 

**Figure 3:**
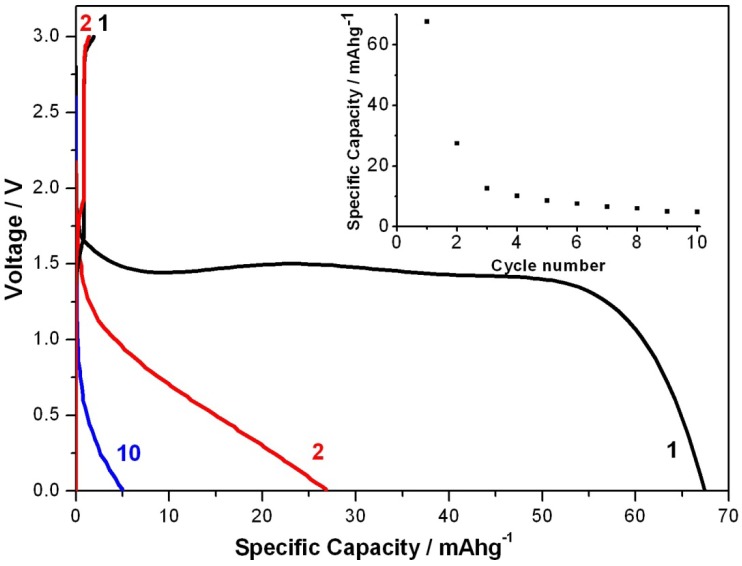
Galvanostatic discharge/charge curves of the textile battery at 1/6 C between 0 and 3 V in coin cell set up. The change of specific capacities with cycle numbers is plotted in the right inset of the voltage profile.

To study further electrochemical properties of this type of battery a standard 2032 coin cell was also assembled inside an mBraun glovebox (H_2_O < 2 ppm, O_2_ < 10 ppm). The coin cell was configured in the same manner as shown in Figure 2a, where the PEDOT textile is used as the cathode, the lithium foil as an anode and poly(ethylene glycol) borate ester is the polymer electrolyte. The coin cell was discharged with a Maccor battery tester at different current densities ranging from of 10 mA/g (1/6 C) to 240 mA/g (4 C). Figure 3 shows the galvanostatic discharge/charge voltage profile of such coin cell with the textile PEDOT electrode. 

The cell was firstly cycled at 1/6 C for 10 cycles. The 1st, 2nd and 10th cycle of discharging curves are shown in Figure 3. Specific capacity for all-solid-state Li/polythiophene is about 44 mAh/g [18]. Batteries based on cellulose and polypyrrole exhibited charge capacities between 38 and 50 mAh/g per weight of the active material [17], which was the highest charge capacity reported for all conducting polymer based batteries. In contrast, the energy capacity for our textile PEDOT battery is 68 mAh/g during the first discharge, which is higher than the highest value reported to date for all-solid conducting polymer batteries. During the first discharging process the voltage reaches a plateau at about 1.5 V. Specific capacity decreased to about 27 mAh/g during the second discharge and was stabilized at about 5 mAh/g at the 10th cycle. Change of specific capacity was shown in the insert in Figure 3. Even though the energy capacity decreases during discharging, the initial discharge capacity is the highest of its kind for primary PEDOT batteries. It should also be noticed that the battery is very quickly recharged and it only took about 7 min to be fully charged at 1/6 C. Different charging rates have been tried. When discharged with 4 C, the charging time was decrease further from 7 min (at 1/6 C) to about 16 s. These results are in good agreement with the fast-charging ability of batteries based on conducting polymers [16]. 

**Figure 4:**
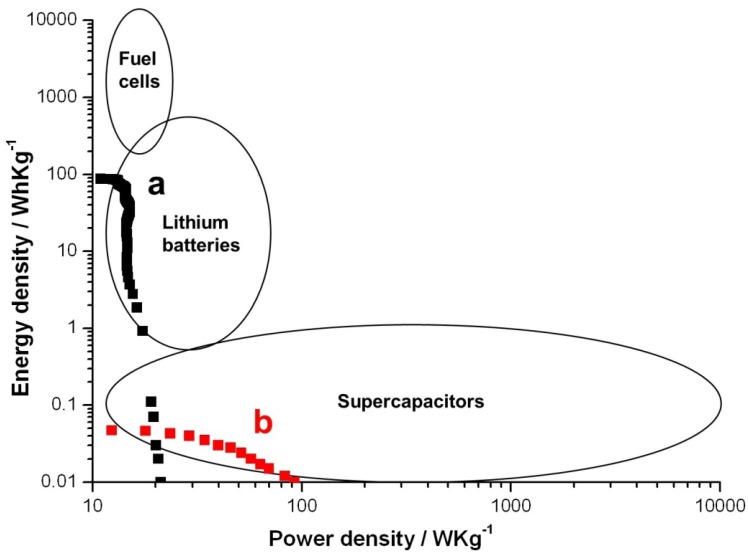
Ragone plots of the textile batteries when discharged at (**a**) 1/6 C and (**b**) 4C when using the PEDOT textile battery as a primary battery.

The specific energy of lithium ion batteries is typically on the order of 10–300 Wh/kg, but the specific power is only on the order of 1–100 W/kg. The typical specific energy and power of supercapacitors are in the order of 0.01–1 Wh/kg and 10–1000 W/kg, respectively. These specific values of supercapacitors are not high enough to replace batteries in many applications. A Ragone plot of the textile battery is shown in Figure 4 with the operating ranges of fuel cells, lithium batteries and super capacitors outlined. When the battery was discharged at 1/6 C, it behaves like a typical lithium battery, and the highest specific energy density was 91 Wh/kg as shown in Figure 4a. However, if the discharge current was increased to 4 C, it behaves like atypical supercapacitor with highest specific power density of 100 W/kg. Depending on the discharging current, this device can be used both as a battery and a supercapacitor. 

## 3. Experimental Section

The polyester/cellulose hydroentangled nonwoven fabric, “Contec amplitude EcoCloth” was purchased from Fisher Ltd and lithium metal foil was purchased from SigmaAldrich Ltd. Highly conductive PEDOT:PSS nano-emulsion ink, which can be directly used in printing applications was supplied by DKSH Ltd. 

Standard 2032 coin cell was assembled inside an mBraun glovebox filled with inert Ar gas. The electrochemical properties of the assembled coin cell were studied with a Maccor battery tester.

## 4. Conclusions

In conclusion, by impetrating high-surface area textiles with nano-emulsion conducting polymer inks, we solved the challenge of loose contact and adhesion problems when applying conducting polymers in batteries. It converts the insulating textile fabric to conductive electrode of a lithium battery with the highest specific energy capacity of 68 mAh/g, which is superior to most of the solid-state conducting polymer primary and secondary batteries reported. Reliable, flexible and rollable textile batteries can be made by this method and are suitable for wearable/woven electronics. Such a battery can be quickly recharged in 7 min at 1/6 C and in 16 s at 4 C. This battery also enables a new and desirable portfolio of textile products which could be used as both decoration accessories and energy sources at the same time, for example, a heart rate/blood pressure monitor with textile power supplies for conformable clothing, or a phone sock made from textile batteries to charge mobile devices wirelessly. 
